# Storage conditions of high‐fat diets affect pulmonary inflammation

**DOI:** 10.14814/phy2.15116

**Published:** 2021-11-25

**Authors:** Marta Kokoszynska, Niki D. Ubags, Joseph J. Bivona, Sebastian Ventrone, Leah F. Reed, Anne E. Dixon, Matthew J. Wargo, Matthew E. Poynter, Benjamin T. Suratt

**Affiliations:** ^1^ Department of Medicine Pulmonary Disease and Critical Care Medicine University of Vermont Larner College of Medicine Burlington Vermont USA; ^2^ Vermont Lung Center Burlington Vermont USA; ^3^ Faculty of Biology and Medicine University of Lausanne Service de Pneumologie CHUV Lausanne Switzerland; ^4^ Cellular, Molecular, and Biomedical Sciences Doctoral Program University of Vermont Burlington Vermont USA

## Abstract

Obesity alters the risks and outcomes of inflammatory lung diseases. It is important to accurately recapitulate the obese state in animal models to understand these effects on the pathogenesis of disease. Diet‐induced obesity is a commonly used model of obesity, but when applied to other disease models like acute respiratory distress syndrome, pneumonia, and asthma, it yields widely divergent. We hypothesized high‐fat chow storage conditions would affect lipid oxidation and inflammatory response in the lungs of lipopolysaccharide (LPS)‐challenged mice. For 6 weeks, C57BL/6crl mice were fed either a 10% (low‐fat diet, LFD) or 60% (high‐fat diet, HFD) stored at room temperature (RT, 23°C) for up to 7, 14, 21, or 42 days. Mice were treated with nebulized LPS to induce lung inflammation, and neutrophil levels in bronchoalveolar lavage were determined 24 h later. Lipid oxidation (malondialdehyde, MDA) was assayed by thiobarbituric acid reactive substances in chow and mouse plasma. Concentrations of MDA in chow and plasma rose in proportion to the duration of RT chow storage. Mice fed a HFD stored <2 weeks at RT had an attenuated response 24 h after LPS compared with mice fed an LFD. This effect was reversed after 2 weeks of chow storage at RT. Chow stored above freezing underwent lipid oxidation associated with significant alterations in the LPS‐induced pulmonary inflammatory response. Our data show that storage conditions affect lipid peroxidation, which in turn affects pulmonary inflammatory responses in a mouse model of disease. It also suggests changes in the microbiome, although not significantly different suggests decreased variety and richness of bacteria in the gut, a large aspect of the immune system. Dietary composition and storage of chow may also affect pulmonary inflammation and the gut microbiome in humans.

## Graphical abstract

Storage conditions of mouse chow affect lipid perodixation of chow, which in turn affects inflammatory responses in the lungs. This may explain the divergent results seen in acute lung injury models using diet‐induced obesity. Data analysis may be affected simply because of the state of mouse chow.

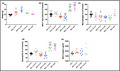

## 1 INTRODUCTION

Obesity has become a growing threat to human health. It affects the incidence, manifestations, and outcomes of many diseases (Peters et al., 2018) including asthma and acute respiratory distress syndrome (ARDS; Stapleton & Suratt, 2014). Obesity itself is a complex state associated with varying degrees of metabolic dysfunction caused by a myriad of altered dietary compositions. The multitude of effects that this state has on the body makes it challenging to understand how the syndrome of obesity affects respiratory diseases.

Mouse models are often used to probe how individual facets of obesity and the metabolic syndrome affect lung disease pathogenesis. Among many aspects of the metabolic syndrome, dyslipidemia is a key factor leading to immune dysfunction (Gowdy & Fessler, 2013). It is, therefore, important to accurately and consistently simulate this state in animal models to better understand how it may alter the course of a concomitant lung disease, such as ARDS. One of the most commonly used models of obesity and dyslipidemia is diet‐induced obesity (DIO), in which mice are fed high‐fat chow (e.g., 60% fat) for 15–20 weeks. However, widely divergent results have been reported when DIO is superimposed on standard disease models of ARDS and asthma, despite the use of identical diets (Kordonowy et al., 2012; Shah et al., 2015; Ubags et al., 2016). Potential explanations for these divergent results have included subtly differing experimental approaches, vendor‐ or facility‐related alterations in microbiome, and differences between mouse substrains (Rasmussen et al., 2019; Ubags et al., 2016; Wolff et al., 2020). However, we propose another explanation, differences in the dietary composition of oxidized lipids. Oxidation rates of dietary components is affected by the fatty acid composition, level of unsaturated fats, presence and activity of pro‐oxidants/antioxidants, and storage conditions of the foods (temperature and exposure to light and humidity; Kanner, 2007). From cardiac literature, we know that this nutrient oxidation composition plays a role in the regulation of chronic inflammation, leading to increased levels of inflammatory biomarkers and alterations in peripheral blood monocytes (Addis, 1986; Cohn, 2002; Amarjit et al., 2009; Roberts et al., 2018).

We sought to investigate how oxidation of dietary components might affect pulmonary inflammation, as although vendors recommend that high‐fat chow be stored frozen and frequently changed‐out in cages, this may be logistically difficult and expensive. Because of the increased abundance of substrates, high‐fat diet (HFD) chow is more prone to lipid oxidation under nonideal temperatures and light exposure. We hypothesized that storage temperatures above freezing lead to an increase in lipid oxidation of high‐fat chow over time that augments the LPS‐induced pulmonary inflammatory response (a mouse model of ARDS) of mice fed such chow. Our results have implications for both mouse models of respiratory disease and may be pertinent to the ever changing diet of people and how it affects human disease as well.

## 2 MATERIALS AND METHODS

### 2.1 Mice

Twelve‐week‐old, female, C57BL/6crl mice were purchased from The Charles River Laboratory and housed in an American Association for the Accreditation of Laboratory Animal Care‐approved facility at the University of Vermont, maintained on a 12 h light/dark cycle, and provided food and water ad libitum. Mice were placed on a HFD (60% fat; D12492; Research Diets) to create DIO, while controls were placed on an low‐fat (10% fat) chow diet (D12451; Research Diets). The recommendations from the manufacturer for the 60% diets are as follows: “Most diets require storage in a cool dry environment. Stored correctly they should last 3–6 months. Because of the high fat content is best if kept frozen.” The diet study was started at 12 weeks of age and continued for 6–8 weeks. The diets were kept either frozen (−20°C) or refrigerated (4°C) in a closed, airtight, opaque container inside and refrigerator and freezer dedicated just to that food without exposure to light. The mice were fed the food directly and it was changed once weekly. The food was aged at room temperature (RT; 23°C) and placed in cages with mice for the varying durations exposed to the same light/dark cycle as the mice. Food in cages was replaced once in a week. The diets for all experiments were used within a similar time frame of expiration. Two different lot numbers were used with fat between chow groups being identical. There were five mice per cage maximum, and the mice were able to consume the food in the hoper ad libitum. Experiments were performed in accordance with the Animal Welfare Act and the USPHS Policy on Humane Care and Use of Laboratory Animals after review by the Animal Care and Use Committee of the University of Vermont.

### 2.2 LPS‐induced lung inflammation

Mice were exposed to aerosolized *Escherichia coli* 0111:B4 LPS (Sigma) PMID 17548653, PMID 24231757) for 15 min (de Souza Xavier Costa et al., 2017). The mice were euthanized with sodium pentobarbital (150 mg/kg by i.p. injection; Wilcox Pharmacy) overdose and exsanguination 24 h after LPS exposure. Plasma, bronchoalveolar lavage (BAL) fluid, and whole lung were collected. Samples were snap‐frozen in liquid nitrogen and BAL cells were scored for total and differential cell counts.

### 2.3 RNA isolation and quantitative real‐time polymerase chain reaction

Whole lung tissue (~100 mg) was homogenized using mortar and pestle and subsequently placed within gentle MACS M Tubes (Miltenyi Biotec) containing 500 μl of phosphate‐buffered saline and protease inhibitor cocktail (MilliporeSigma) and lysed on a gentleMACS Dissociator according to manufacturer's settings. Lysate was then passed through QIAshredder spin columns (Qiagen). The concentration and quality were measured using a NanoDrop 2000 spectrophotometer (Thermo Fisher Scientific). cDNA was synthesized from 200 ng of RNA using the qScript Supermix reagent kit per manufacturer's instructions (Quantabio). Quantitative real‐time PCR was performed using iTaq Universal SYBR Green Supermix on a CFX96 Touch (Bio‐Rad Hercules), with the relative mRNA expression calculated using the threshold cycle (Ct; 2^−ΔΔCt^) method normalized to *Gapdh* expression. Primers were designed for mouse *Il6* (5′‐CCGGAGAGGAGACTTCACAG‐3′ and 5′‐GAGCATTGGAAATTGGGGTA‐3′), *Ccl2* (MCP‐1) (5′‐GAGCTACAAGAGGATCACCAGCA‐3′ and 5′‐GTTCTGATCTCATTTGGTTCCGATCC‐3′), *Icam1* (5′‐GAGCCAATTTCTCATGCCGC‐3′ and 5′‐TCGAGCTTTGGGATGGTAGC‐3′), *Vcam1* (5′‐CTGGGAAGCTGGAACGAAGT‐3′ and 5′‐GCCAAACACTTGACCGTGAC‐3′), *Cxcl1* (5′‐GCTGGGATTCACCTCAAGAA‐3′ and 5′‐TGGGGACACCTTTTAGCATC‐3′), *Tnf* (5′‐TCCCAGGTTCTCTTCAAGGGA‐3′ and 5′‐GGTGAGGAGCACGTAGTCGG‐3′), *Tlr4* (5′‐AAATGCACTGAGCTTTAGTGGT‐3′ and 5′‐TGGCACTCATAATGATGGCAC‐3′), *Ly6g* (5′‐TGTATTGGGGTCCCACCTGA‐3′ and 5′‐TGGGAAGGCAGAGATTGCTC‐3′), and *Cxcl5* (5′‐TCCTCAGTCATAGCCGCAAC‐3′ and 5′‐TGCGAGTGCATTCCGCTTA‐3′). Quantitative RT‐PCR was performed using SYBR Green Supermix (Bio‐Rad) and normalized separately to *Gapdh* (5′‐ACGACCCCTTCATTGACCTC‐3′ and 5′‐TTCACACCCATCACAAACAT‐3′).

### 2.4 Protein quantification by enzyme‐linked immunosorbent assays and Luminex multiplex

Levels of IL‐17A and G‐CSF were quantified using DuoSet ELISA kits according to the manufacturer's protocol (R&D Systems). Absorbance was read at 570 nm, and background was removed using 450‐nm absorbance on a Synergy HTX plate reader and Gen5 software (BioTek).

### 2.5 Malondialdehyde quantification

Oxidation was determined by thiobarbiturate acid–reactive substances (TBARS) assay (Oxitech; Zeptometrix), which was then normalized to protein concentration. The level of plasma and chow oxidation was determined using heparin/manganese precipitation (Bohm et al., 2013) followed by protein quantification and TBARS assay, as above.

### 2.6 Fecal microbiome analysis

Fecal pellets were freshly collected at 6–8 weeks after the start of the diet, and samples were stored at −80°C. Bacterial DNA was extracted using Qiagen kit according to manufacturer's instructions as described previously (Wen & Duffy, 2017). Standards were generated from the pooled products of mixed mouse fecal samples following 20 rounds of PCR, which were subsequently used to establish standard curves for each of the primer reactions over a range of fourfold dilutions between 1:100 and 1:400,000. Cycle threshold (Ct) values for each PCR reaction were applied to the respective standard curve, run on each plate on a Bio‐Rad CFX96 or Chromo4 96‐well quantitative real‐time PCR detection system. PCR product abundance was log‐transformed and the average abundance in the LFD group was used to normalize relative abundance from each animal, values that were then used to calculate the mean ± SEM for each bacterial 16S rRNA in each mouse group. A 5 ng input per sample into the NextFlex 16S V4 Amplicon Sequencing kit was used to prepare the libraries per manufacturer's protocol. (https://perkinelmer‐appliedgenomics.com/home/products/library‐preparation‐kits/16s‐18s‐rrna‐sequencing/nextflex‐16s‐v4‐amplicon‐seq‐kit‐2‐0/). 16S rRNA sequences were processed and analyzed using Quantitative Insights into Microbial Ecology (v1.9.0) software. Merging of paired forward and 275 reverse reads was performed using fastq‐join before demultiplexing and quality filtering (quality Phred 276 score *Q* > 20, <3 low‐quality base calls). Operational taxonomic units (OTUs) were assigned using a 277 closed OTU picking strategy with Uclust at 97% identity against the 97% Greengenes reference 278 database (v13.5). Visualization and analysis of OTU tables was performed using Genocrunch (v2; https://genocrunch.epfl.ch).

### 2.7 Data analysis and statistics

Data were analyzed using Graphpad Prism 8 (Graphpad Software). Data are presented as mean ± SEM. Data from each experiment were confirmed by two or more replicative experiments. Experimental groups were compared using one‐ and two‐way ANOVA with appropriate post hoc tests, which are indicated in figure legends.

## 3 RESULTS

### 3.1 High‐fat chow storage conditions alter the pulmonary inflammatory response in LPS‐injured mice

**FIGURE 1:**
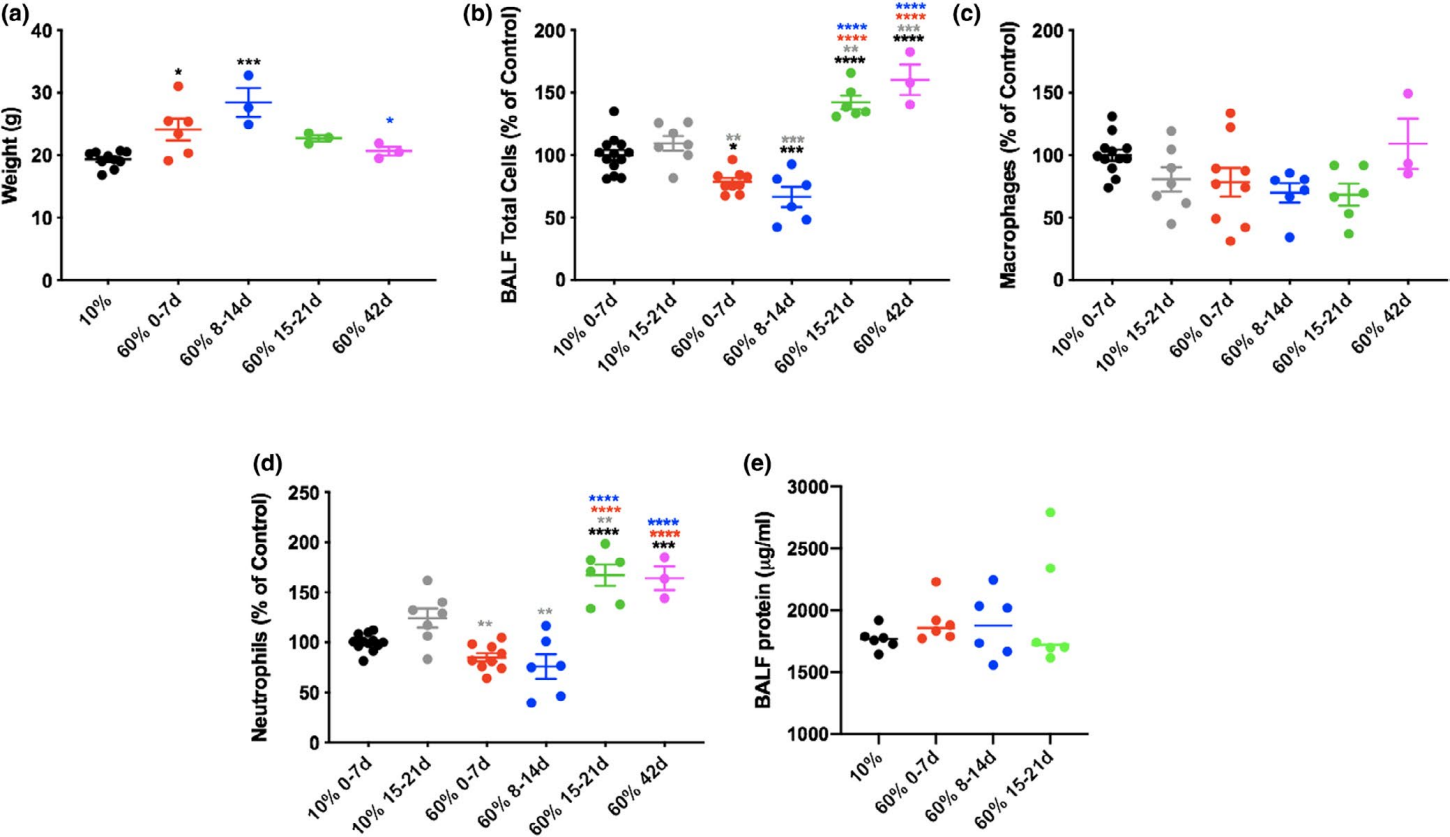
Duration of room temperature storage of 60% fat chow affects weight and pulmonary inflammatory response to LPS. Mice were fed either 10% fat chow or 60% fat chow for 6 weeks that had been stored at room temperature for the durations noted in the figure before being exposed to nebulized *Escherichia coli* LPS O111:B4 (3 mg/ml; 15 min). 24 h later, mice were weighed (a) before being euthanized and bronchoalveolar lavage performed for cell count, differentials and protein (b–e). *n* = 3–6 mice per group *p ≤ 0.05; **p ≤ 0.01; ***p ≤ 0.001; ****p ≤ 0.0001 compared with groups indicated by colored stars/asterisks

To determine how high‐fat (60%) chow storage conditions alter the pulmonary inflammatory response, mice were maintained for 6–8 weeks on either low‐fat (10%) chow or high‐fat chow stored at RT (23°C) for 0–7, 8–14, 15–21, or 37–42 days after initial storage at −20°C. All mice on the HFD demonstrated significant difference in weight compared with those on the LFD, except for those fed chow that had been aged 37–42 days at RT. The end weight on the high‐fat chow was variable, peaking in the group fed chow aged 8–14 days, and progressively decreased in groups fed chow that had been aged for longer periods (Figure 1a). There was a dichotomous effect on the lung inflammatory response to nebulized LPS based on the age of the chow:mice fed high‐fat chow stored at RT for less than 14 days demonstrated lower airspace neutrophil levels compared with those fed LFD, whereas those fed high‐fat chow stored at RT greater than 14 days demonstrated much higher neutrophil levels (Figure 1d). The pattern did not mimic the levels of protein, but the levels of protein did confirm lung injury in all groups noted (Figure 1e). Airspace macrophage levels were not significantly altered by the storage duration of the chow (Figure 1b).

**FIGURE 2:**
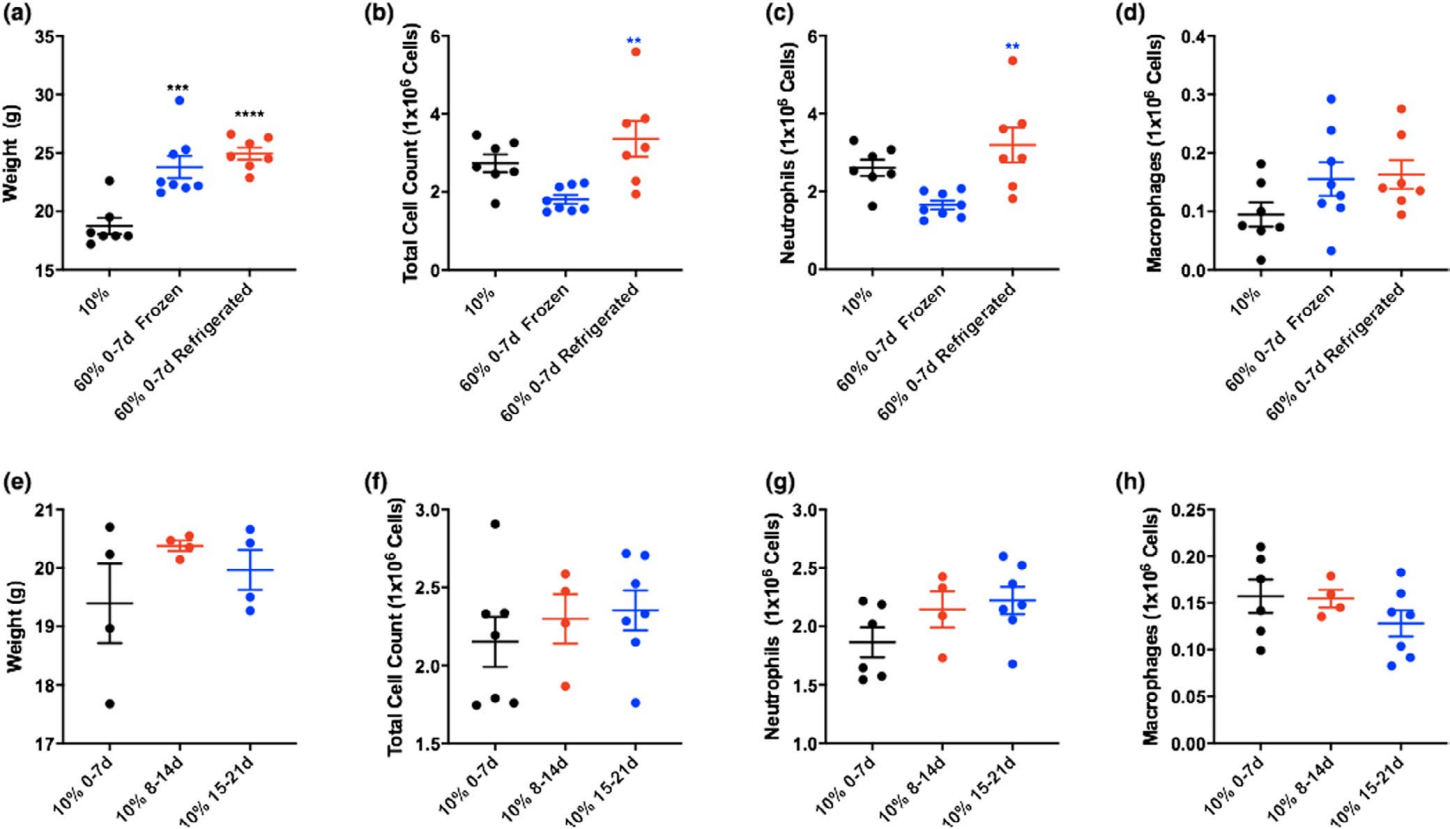
Storage of 60% chow at 4°C increases inflammation compared with chow stored at −20°C. Mice were fed for 6 weeks with (10% 0–7 days) fat diet versus 60% (0–7 days) fat diet frozen (−20°C) or stored at 4°C (refrigerated) for 6 weeks, then challenged with LPS. Weights (a) and bronchoalveolar lavage cell counts and differentials (b–d) are shown. *n* = 7 mice in 10%, *n* = 8 mice in 60% frozen and *n* = 7 mice in 60% refrigerated groups. Storage of 10% chow at room temperature (RT) does not alter the weight (e) of pulmonary inflammatory response in mice fed such chow (f–h). Mice were fed for 6 weeks with 10% fat chow that had been stored at RT for the durations noted in the figure before injury. 24 h later, mice were weighed (e) (*n* = 4 mice in 10% 0–7, *n* = 4 mice in 10% 8–14, *n* = 4 mice in 10% 15–21 groups) before being euthanized and bronchoalveolar lavage performed for cell counting and differentials (f–h) *n* = 6 mice in 10% 0–7, *n* = 4 mice in 10% 8–14, *n* = 7 mice in 10% 15–21 groups. *p ≤ 0.05; **p ≤ 0.01; ***p ≤ 0.001; ****p ≤ 0.0001 compared with group indicated by colored stars/asterisks

We next determined how refrigeration affected weight and airway inflammation. Both groups of mice on refrigerated and frozen high‐fat chow had significantly higher weights compared with the mice on low‐fat chow, but there was no difference in weight between the mice on the refrigerated and frozen chow. There was a significantly increased inflammatory response to nebulized LPS in the mice fed refrigerated compared with frozen high‐fat chows (Figure 2a–d).

### 3.2 Storage conditions of low‐fat (10%) diets have minimal influence on the pulmonary inflammatory response in mice

To better understand whether the lipid content, which is higher in high‐fat chow compared with low‐fat chow, may be playing a role in this change to inflammatory response we examined LFD that had been stored at RT for similar durations to the high‐fat chow in Figure 1. Although there was a trend toward higher cell counts in mice fed 10% chow stored at RT for greater durations (Figure 2e–h), there were no significant differences.

### 3.3 High‐fat chow storage conditions are associated with alterations in cytokine response in LPS‐injured mice

**FIGURE 3:**
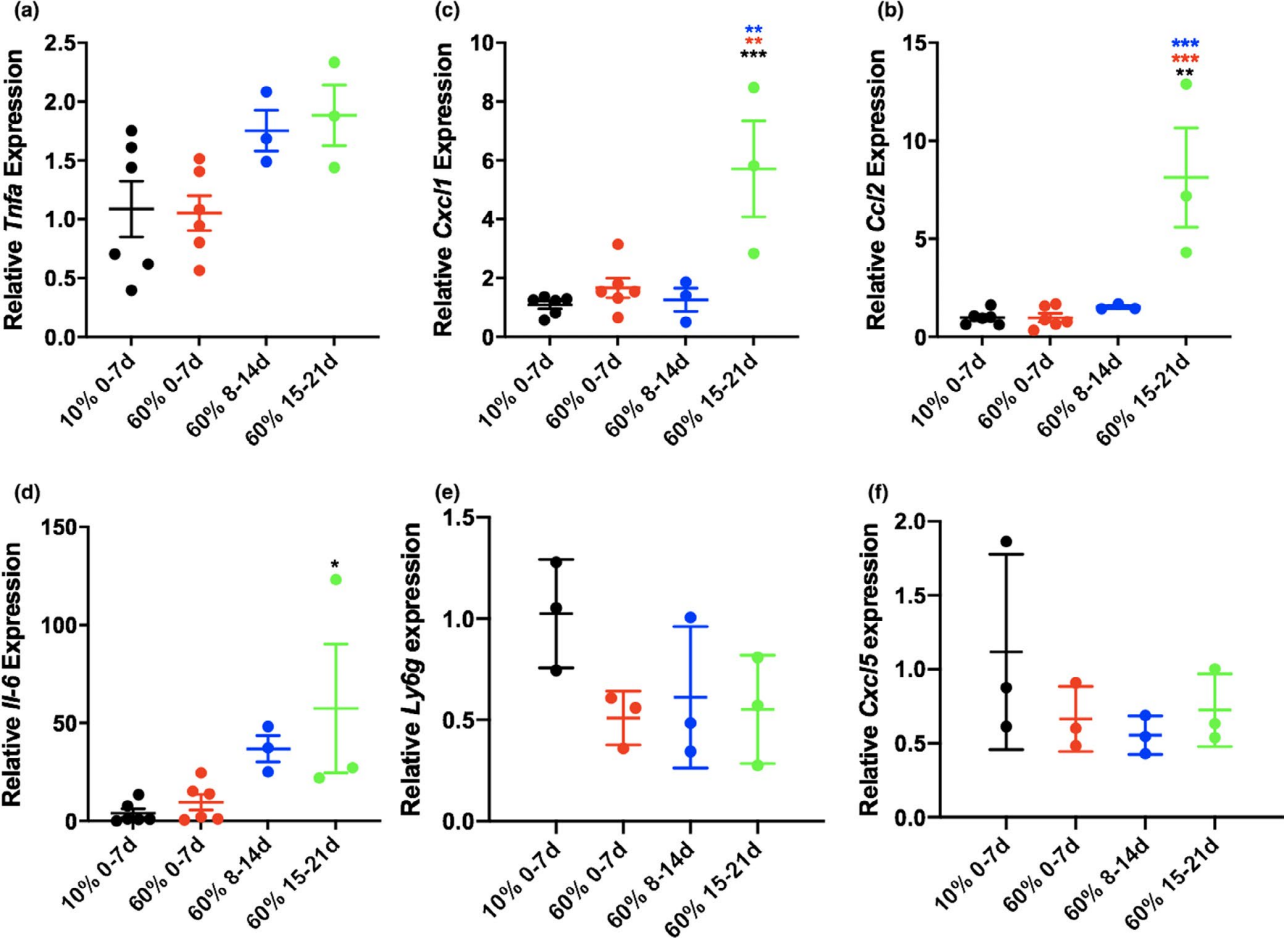
Cytokine response is augmented with age of chow at room temperature. Whole lung cytokines (a–d) levels 24 h after nebulized LPS exposure in lean compared with diet‐induced obese mice were determined by quantitative reverse transcription PCR. TNFa, tumor necrosis factor alpha; *Ccl2*, chemokine ligand 2; *Cxcl1*, chemokine ligand 1; *Il‐6*, interleukin 6; *Ly6g*, lymphocyte antigen 6 complex locus G6D; *Cxcl5*, C‐X‐C Motif Chemokine Ligand 5. *N* = 6 mice in 10% 0–7, *n* = 6 mice in 60% 0–7, *n* = 3 mice in 60% 8–14, *n* = 3 mice in 10% 15–21 groups.*p ≤ 0.05; **p ≤ 0.01; ***p ≤ 0.001; ****p ≤ 0.0001 compared with group indicated by colored stars/asterisks

We next sought to determine if pulmonary inflammatory cytokine expression in response to LPS lung injury was also altered by high‐fat chow storage conditions at RT. There were significantly higher levels of *Ccl2* (MCP‐1), *Cxcl1* (KC) and *Il6* (IL‐6) in whole lung of mice fed 15–21 day old high‐fat chow compared with either low‐fat chow or high‐fat chow aged for a shorter length of time. *Tnf* (TNFα), Ly6G and Cxcl5 expression did not differ between groups (Figure 3).

### 3.4 High‐fat chow storage conditions do not alter baseline cellular adhesion molecules or cellular response in the lung

**FIGURE 4:**
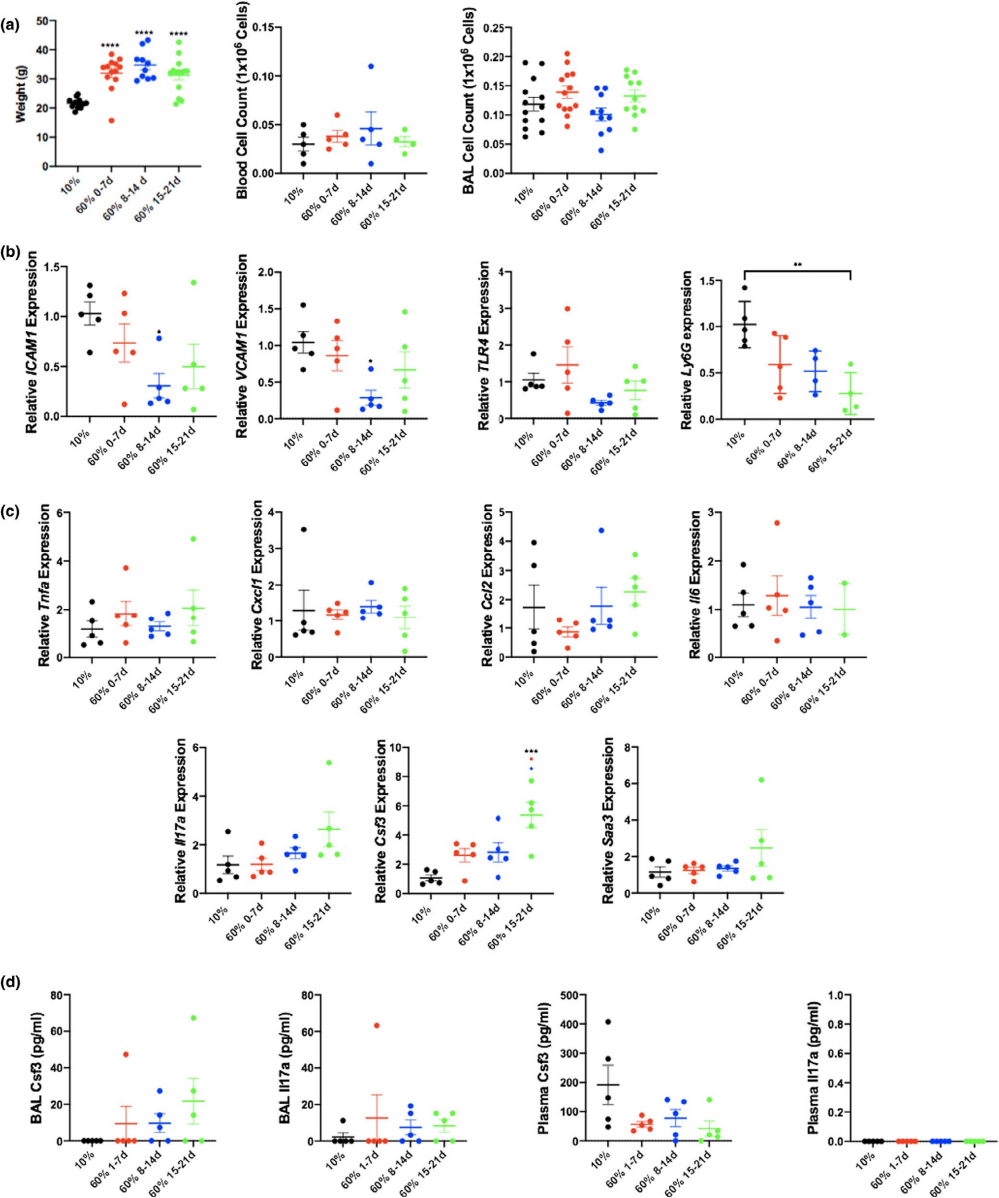
Uninjured mice fed 60% chow show significant increase in weights but no difference in bronchoalveolar lavage cell counts. Weights were recorded before mice were euthanized and bronchoalveolar lavage fluid and blood total cell counts were measured (a) (weights *N* = 13 mice in 10% 0–7, *n* = 13 mice in 60% 0–7, *n* = 10 mice in 60% 8–14, *n* = 14 mice in 60% 15–21 groups; plasma *N* = 5 mice in 10% 0–7, *n* = 5 mice in 60% 0–7, *n* = 5 mice in 60% 8–14, *n* = 4 mice in 60% 15–21 groups; BAL *N* = 13 mice in 10% 0–7, *n* = 13 mice in 60% 0–7, *n* = 10 mice in 60% 8–14, *n* = 11 mice in 60% 15–21 groups). Exposure to high‐fat chow kept at room temperature does not increase mouse lung VCAM‐1, ICAM, TLR4 expression at baseline, but did decrease Ly6G between 10% and 60% 15–21 (b) *N* = 5 in all groups. *p < 0.05 compared with 10% diet group. There was no significant differences in inflammatory cytokine transcription at baseline between groups except for G‐CSF (c) There was no difference in the levels of cytokines (G‐CSF and IL‐17A) transcribed in blood or BAL (d) (*N* = 5 mice in 10% 0–7, *n* = 5 mice in 60% 0–7, *n* = 5 mice in 60% 8–14, *n* = 5 mice in 60% 15–21 groups). **p ≤ 0.01; ***p ≤ 0.001; ****p ≤ 0.0001 compared with group indicated by colored stars/asterisks

We examined our model of chow aged at RT for different time points on uninjured mice to determine whether the same variable effect in inflammation is also present. All mice on the HFD demonstrated significant difference in weight compared with those on LFD (Figure 4a). We previously demonstrate that the most significant change in the cellular population of the pulmonary innate system was in the number of neutrophils found in the lungs of mice fed older chow. We examined the role of cellular adhesion molecules, the major mechanism of neutrophil migration into the lung. VCAM‐1 protein mediates the adhesion of lymphocytes to vascular endothelium. It also functions in leukocyte‐endothelial cell signal transduction and increased gene transcription in response to TLR4 stimulation. Similarly, ICAM‐1 is induced by TNFα and is expressed by the vascular endothelium and lymphocytes leading to leukocytes binding to endothelial cells leading to transmigration into tissues (Roberts et al., 2018). We found that there is no increase in VCAM‐1, ICAM or TLR4, suggesting that these are likely not playing a significant role in the alterations seen in the cellular milieu (Figure 4b). However, we say a significant decrease in Ly6G from 10% to oldest 60% chow showing that there may not be an increased number of circulating neutrophils but increased migration in the setting of injury despite nonsignificant Cxcl5 changes (Figure 4b). Additionally, uninjured mice fed aged high‐fat chow showed no significant change in the transcription levels of most inflammatory cytokines at baseline. This was confirmed at the protein level, which indicated no differences in the secretion of inflammatory cytokines at baseline (data not shown). G‐CSF expression levels were significantly increased in the lungs of mice fed older high‐fat chow; however, there were no significant differences in G‐CSF protein levels in the plasma or BAL (Figure 4c,d). Similar results were obtained for IL‐17A levels.

### 3.5 Storage conditions of chow do not affect the gut microbiome in mice

**FIGURE 5:**
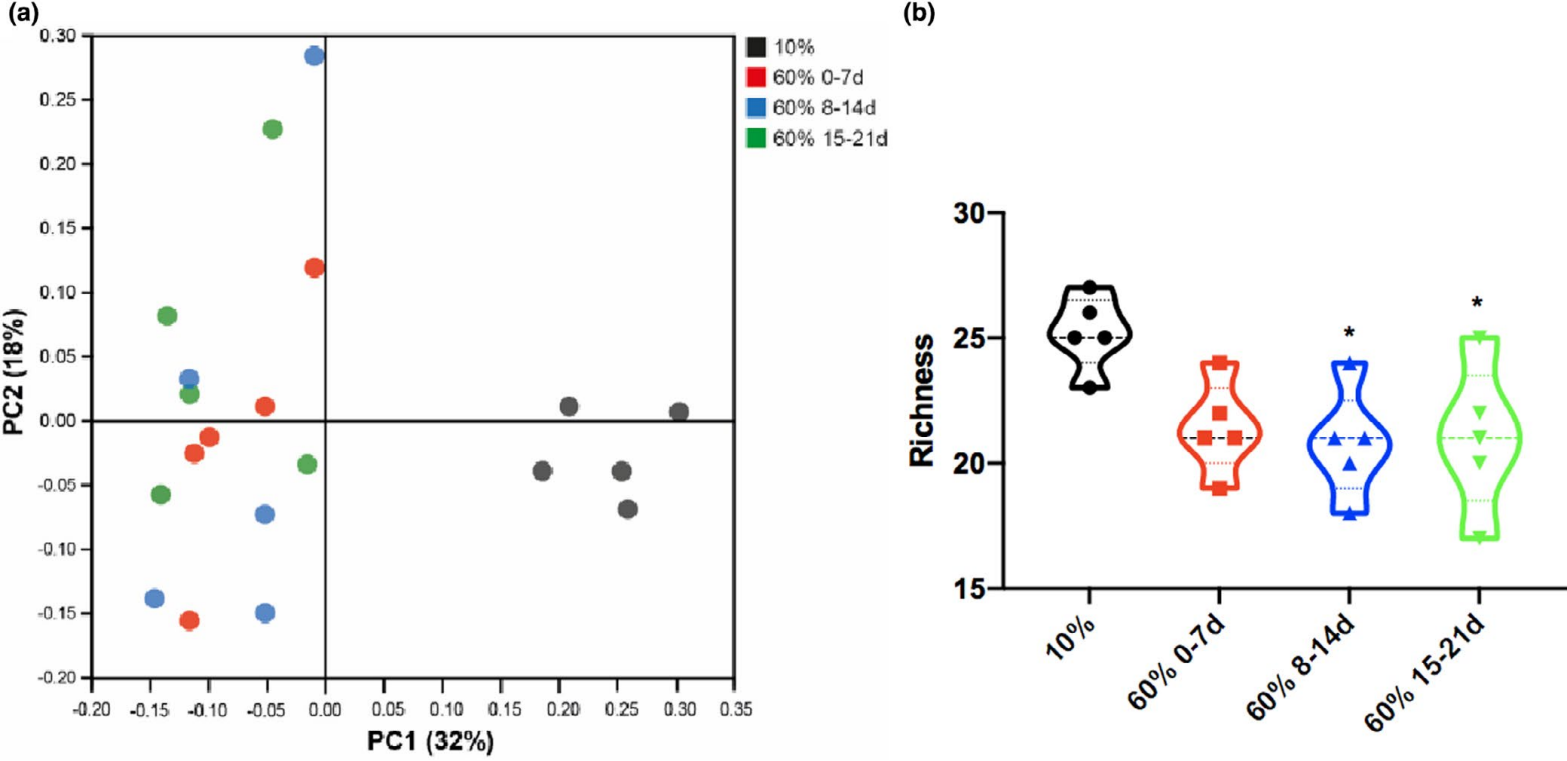
Mice fed aged chow show no change in the gut microbiome in acute lung injury. There was a difference in the microbiome between 10% and 60% fat diets (a) and a significantly decreased level of richness of the microbiome in older chow (b). Bacterial DNA was extracted from stool samples (Qiagen), 16S rRNA gene amplicon library was prepared and 16S rRNA sequencing was performed. (a) Principal‐coordinate analysis plot (based on Bray–Curtis distance) of gut microbiome composition. (b) Microbial richness for each sample was calculated. *n* = 5 per group *p ≤ 0.05 compared with group indicated by colored stars/asterisks based on one‐way ANOVA

Different diets affect the bacterial community composition of the gut. We used our model to examine the fecal bacterial composition using 16S rRNA sequencing in LPS‐injured mice to better understand the spectrum of changes that may occur with aged chow. Principal‐coordinate analysis of fecal samples from mice on the different diets indicate that the gut microbiome of mice on high‐fat chow with different storage conditions is more similar than the gut microbiome of mice fed high‐fat chow compared with low‐fat chow (Figure 5a). Moreover, richness was higher in feces from mice fed a low‐fat chow compared with a high‐fat chow independent of the storage conditions (Figure 5b). These data indicate that storage conditions of high‐fat chow do not significantly affect the gut microbiota.

### 3.6 Storage conditions affect high‐fat chow and plasma oxidation

**FIGURE 6:**
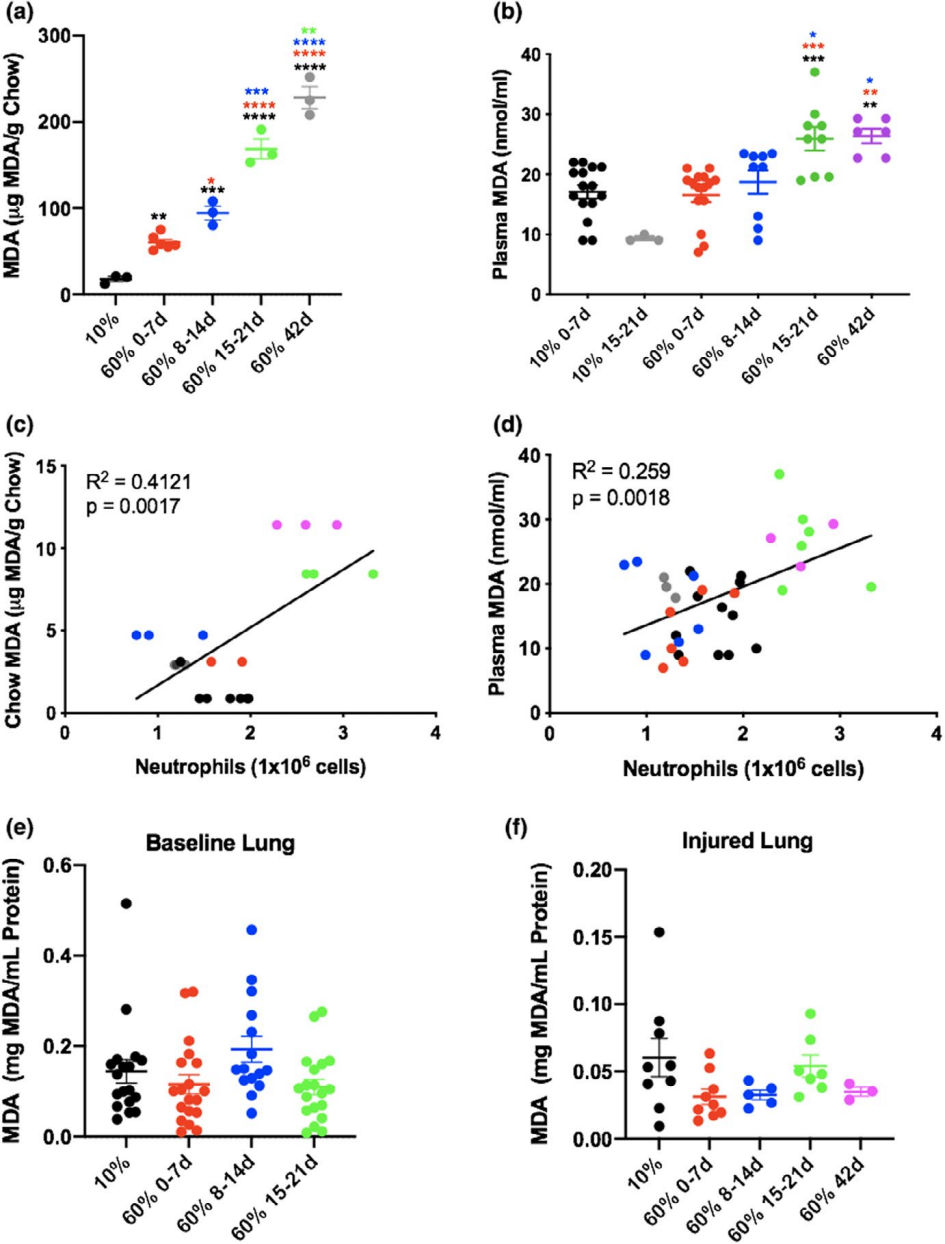
Mice were exposed to nebulized *Escherichia coli* LPS (3 mg/ml; 15 min) 24 h before undergoing bronchoalveolar lavage (BAL) and blood draws. Levels of MDA (lipid oxidation) were measured in plasma in each group using thiobarbiturate acid–reactive substances (TBARS) (a). BAL neutrophil levels were compared with plasma and chow MDA, respectively (b, c). Panels (a) and (b) *N* = 15 mice in 10% 0–7, *n* = 3 mice in 60% 0–7, *n* = 15 mice in 60% 8–14, *n* = 9 mice in 60% 15–21 *n* = 6 mice in 60% 42 groups. Panel (c) *N* = 3 food samples per group. **p ≤ 0.01; ***p ≤ 0.001 compared with the group with matching color. Exposure to high‐fat chow kept at room temperature had no effect on the MDA levels seen in whole lung at baseline and with LPS (d, e). Whole lung tissue (~100 mg) was homogenized using mortar and pestle and levels of MDA (lipid oxidation) were measured in whole lung using TBARS assay. Baseline *N* = 18 mice in 10% 0–7, *n* = 19 mice in 60% 0–7, *n* = 15 mice in 60% 8–14, *n* = 19 mice in 60% 15–21 groups. Injured *N* = 9 mice in 10% 0–7, *n* = 9 mice in 60% 0–7, *n* = 5 mice in 60% 8–14, *n* = 7 mice in 60% 15–21, *n* = 3 mice in 60% 42 groups. Room temperature storage of 60% fat chow leads to increasing levels of oxidized lipids over time (f). Levels of MDA (lipid oxidation) were measured in chow using TBARS assay. Three pellets per group. *p ≤ 0.05; **p ≤ 0.01; ***p ≤ 0.001; ****p ≤ 0.0001 compared with the group with matching color

Food high in fat has the potential to become oxidized, especially when it is stored above freezing and exposed to light and moisture. Measurement of MDA (a marker of lipid oxidation) in high‐fat chow using TBARS assay revealed significantly increased the levels of oxidized lipids in proportion to the duration of time the food had been stored at RT (Figure 6a). These oxidized lipids could provide a dietary source of lipid peroxides that would be reflected in the mice in vivo.

### 3.7 Increasing the duration of storage of high‐fat chow at RT leads to higher levels of oxidation in the plasma of mice fed this chow, which are strongly associated with the altered inflammatory response

Oxidation of dietary lipids affects the composition of circulating lipids. Plasma oxidation was measured by TBARS in samples taken from LPS‐injured mice fed 10% fat chow versus 60% fat chow that had been stored at RT up to 42 days. A significant rise in plasma oxidation was seen with an increasing duration of RT 60% fat chow storage (Figure 6b). Furthermore, airspace neutrophil levels in these mice correlated with both plasma and chow oxidation (Figure 6c,d). Plasma MDA was increased, but there was no difference found in MDA levels in whole lung at baseline and with LPS (Figure 6e,f).

## 4 DISCUSSION

We have shown that mouse models of obesity and ARDS are sensitive even to minor changes in their environment and experimental protocols. Specifically, the conditions under which HFD is stored significantly affect the pulmonary innate immune response to LPS as reflected by the cellular composition in the lavageable airspaces and cytokine expression levels in the whole lung. To better understand this, we explored some possible causes of these varied results through factors in the chow and signaling changes. This not only has an important impact on the way we study human diseases in mouse models and their potential effects in the obese state but also unveiling another component of diets responsible for the unexplained dichotomy seen in diseases like asthma and ARDS in obese versus lean subjects.

Pulmonary innate immunity provides the immediate response to an invading pathogen through physical barriers, complement cascade, cytokines, as well as resident and inflammatory leukocytes such as monocytes, macrophages, and neutrophils (Romero et al., 2015). Changes in this environment alter the response of the pulmonary innate immune system to injury. One example of a population of patients in whom the environment is altered are patients with obesity, a state of low‐grade inflammation that augments responses to different stimuli. Studying these effects is difficult as obesity is not only a factor of enhanced body mass index but also of the metabolic syndrome and all of its components. Therefore, when different lung injury models are applied, consistency in models themselves is important due to the already many complicating factors taking place and potentially affecting the effects of the pulmonary innate immune system. One such model that is commonly used to achieve obesity in mice is DIO. In this model, mice are placed on a LFD (10% fat) and HFD (60% fat). The mice on HFD gain excessive weight and once they are significantly more obese than their 10% diet‐fed counterparts, they are treated with different forms of injury to study the lung inflammatory response. However, studies using the same HFD composition and feeding duration have recorded diverging conclusions in the levels of cytokines and cellular composition of bronchoalveolar lavage fluid (BALF; Fessler & Parks, 2011; Stapleton & Suratt, 2014; Ubags et al., 2016).

There are aspects of the DIO model that are sensitive to changes that cause differing effects on the pulmonary innate immune system. The role of HFD intake/consumption resulting in obesity have long been implicated in cardiac literature as a contributing factor to the effects it has on atherosclerosis (Addis, 1986; Park et al., 2019). Changes in inflammation, body composition, and lipids themselves cause endothelial damage that over time causes plaque build‐up and cardiac disease (Cohn, 2002). The Western diet, high in fat and increasingly associated with fried food, has become the best studied culprit in the development of cardiac disease (Cohn, 2002). Similarly, obesity and diets with high inflammatory indices have been associated with many pulmonary diseases as well, the most established being asthma, COPD, pneumonia, and ARDS (Kanner, 2007).

In our study, we observed that the cellular population and response seen in the BAL of injured mice fed chow aged at RT was different compared with the response in mice fed a diet that was stored frozen. Aged chow caused a variability in weights, specifically, there is a significant increase in weight up to 60% when mice were fed a chow that was aged 8–14 days, with a significant decline in weight the older the chow became. This could possibly be related to the rancidity of the chow causing it to be less desirable to eat.

There was a significant decline in total BALF cells in mice fed 60% chow aged for one to 2 weeks, compared with normal 10% chow, with a sharp significant increase seen in chow aged >2 weeks compared with 60% food that was <2 weeks old. The fluctuation in total cell counts based on the RT storage duration of the chow, seems to be mostly be driven by neutrophils and there is no change in the number of macrophages. This was not explained by parenchymal margination of neutrophils or changes in the levels of chemoattractants. Unlike our study, others have shown that mice on HFD have an increased number of lung macrophages (Tashiro et al., 2017).

In another study done in rats, oxidized lipid content of mesenteric lymph chylomicrons (CMs) increased when increasing quantities of oxidized lipids were administered intragastrically. These results indicate that oxidized lipids are packaged into CM and absorbed (Kozul et al., 2008). Similar findings were seen in human studies, when subjects were fed a highly oxidized oil, the content in CMs was increased almost 50‐fold leading to an increase in TBARS versus subjects on a diet that contained minimally oxidized oils (Takeda et al., 1984). Because of their large size, CMs are not atherogenic in and of themselves, but CM remnants have been repeatedly implicated in inflammatory processes.

To better understand whether improper storage of food could be a potential underlying factor altering responses we stored the food at different conditions for different periods of time and observed changes in response to injury. Not only are there changes seen in chow aged at RT, but even chow kept under refrigerated conditions. There is a significant difference in chow that has been refrigerated versus frozen, showing a decline in total cell count in chow that was frozen and the increase in chow that was refrigerated compared with normal 10% and no change in the number of macrophages. Interestingly, chow with lower levels of fat showed no difference in mice in terms of weight or in cell type in the aged 10% chow. This confirmed that alterations in the high‐fat chow directly influenced inflammatory response.

Understanding that the storage of high‐fat chow alters the pulmonary innate inflammatory system, we wanted to deduce the potential mechanisms affecting this response. Because there was such an intense response and difference in injured lung, we next determined whether there was any effect on the lung in uninjured mice. Uninjured mice still had a significant difference in weight on regular and aged chow; however, there was no difference in cell counts in the blood or BAL. There was also no difference in the transcription of inflammatory cytokines except for G‐CSF. Suggesting that the change in response that occurs happens only when these cells are stimulated. Therefore, it was necessary to look further at the microenvironment that may be affecting the sensitivity and intensity with which these cells react to injury.

Dietary composition, and in particular, the consumption of a high‐fat‐containing diet, can alter the composition of the gut microbiome, which can consequently affect the immunological tone of an individual (Wypych et al., 2017). In the current study, we observed that gut microbiome composition of mice fed a low‐fat chow differed in community composition and richness from that of mice fed high‐fat chow, which is in line with previous observations (Wen & Duffy, 2017). Interestingly, storage conditions of the high‐fat chow did not affect gut microbiome composition, suggesting that the observed immunological effects are not directly mediated by alterations in the gut microbiome.

The largest component of the diets is fat. Because there was no change seen in the LFD and only in HFD food that was improperly stored, we focused on the possible effects of the environment on chow that is high in fat because it is well known that chow high in fat can oxidize when improperly stored. The higher fat oxidation has been attributed to a larger availability of free fatty acids (FFAs) for oxidation in tissues, which can directly activate TLR4 (Madenspacher et al., 2010), which among other is expressed on neutrophils and macrophages. TLR4 is also activated by LPS leading to the production of a range of pro‐inflammatory mediator, potentially explaining the exaggerated response when these receptors have already been sensitized by FFAs (Fricke et al., 2018). In both macrophages and adipocytes, nonesterified fatty acids stimulate an increase in TLR4 signaling, including an increase in IL‐6 and TNF alpha mRNA expression (Ogino et al., 2015). What we found was that there were increased levels of lipid oxidation in food that was aged compared with 10% and compared with fresh 60%. There was also a significantly higher levels of lipid oxidation seen chow aged for >3 weeks, correlating with higher levels of neutrophils in plasma and chow upon injury.

Alterations in lipid absorption, composition, and environment alter the function, structure, and signals of cells part of the inflammatory response. There have been many debates whether the main contribution of oxidized lipids is due to just in vivo production and whether there is a substantial contribution from oxidized lipids in the diet. Lipid oxidation in food is one of the major degradative processes responsible for the generation of cytotoxic and genotoxic compounds (Ogino et al., 2015). Unsaturated lipids contained in oils, fats, and food undergo oxidation from the oxygen present in room air (Esterbauer, 1993). However, it is the high thermal changes in heating and frying that cause most of the reactions that oxidize the lipids in food and oils. As revealed in studies conducted on rats, chronic feeding of oxidized materials led to growth retardation, increased lipid peroxides in the liver, and metabolic dysfunction (Esterbauer, 1993; Kozul et al., 2008). MDA is, in many instances, the most abundant individual aldehyde that results from lipid peroxidation in foods (Ogino et al., 2015) and is notably established as a culprit that is high in cell toxicity, involved in cancer, caused by DNA damage (Agmon & Stockwell, 2017; Dung et al., 2006; Kozul et al., 2008).

Although there are higher levels of fat oxidation, we observed no differences in lipid oxidation in baseline or injured whole lung, suggesting that potential changes may be coming from circulatory changes (Bohm et al., 2013). Although obesity is known to impair systemic blood vessel function, and predisposes to systemic vascular diseases, its effects on the pulmonary circulation are largely unknown. In a prior study we demonstrated that the lung endothelium from obese mice expressed higher levels of leukocyte adhesion markers and lower levels of cell–cell junctional proteins compared with lean mice. We found that treatment of primary lung endothelial cells with obese serum enhanced the expression of adhesion proteins and reduced the expression of endothelial junctional proteins compared with lean serum. Alterations in pulmonary endothelial cells observed in obese mice were associated with enhanced susceptibility to LPS‐induced lung injury. Restoring serum adiponectin levels reversed the effects of obesity on the lung endothelium and attenuated susceptibility to acute injury. Our work indicated that obesity impairs pulmonary vascular homeostasis and enhances susceptibility to acute injury. Because of the systemic effects obesity has on pulmonary vasculature, we further wanted to examine whether these changes are also altered by lipid oxidation (Kordonowy et al., 2012; Shah et al., 2015).

What is known so far from other studies is that absorption of oxidized lipids leads to higher levels of oxidized lipids in endogenous lipoproteins. In a study conducted in rats, the levels of oxidized lipid in VLDL + LDL directly correlated with the quantity of oxidized lipid in the diet (Bohm et al., 2013). Additionally, studies in humans showed that the fatty acid composition of the diet determines the fatty acid composition of LDL (Fessler & Parks, 2011). This is an important implication as long‐term regulatory signals, as well as specific oxidized fatty acid and phospholipid components of oxLDL, enhance the ability of vascular endothelial cells to express cytokine‐mediated VCAM‐1. We did no observe significant changes in levels of VCAM‐1; however, transcript levels of these regulators may not correlate with their protein levels or cell surface display. It is still not mechanistically clear whether it is circulatory cells and cytokines that interact with the pulmonary immune system through the vascular endothelium that orchestrate this augmented response or whether their signaling changes within the pulmonary environment itself. One provocative theory is that the augmented induction of endothelial receptors for leukocyte migration, such as VCAM‐1, which is stimulated by these fatty acids, may function to exaggerate the influx of neutrophils into the airspace. This hypothesis was supported by higher levels of VCAM‐1 expression in mice fed aged HFD. However, this needs to be further evaluated along with the mechanistic role and implication of macrophages and foam cell signaling (Fessler & Parks, 2011; Staprans et al., 1993).

Implications of nonabsorbed oxidized compounds during their passage through the small and large intestines. In this respect, mucosal cell modifications and interactions with microflora metabolism may also contribute to LPS‐induced pulmonary inflammation.

Lastly, recent data have shown that dietary fat has the ability to significantly affect surfactant function (Wen & Duffy, 2017). Surfactant function and composition play an important role in the stability of the local environment of the pulmonary immune system. This further underlines the importance of diet and the specific macronutrients present in the diet and their effects on our pulmonary system.

In conclusion, we have shown that diet plays a major role in the pulmonary innate immune response. Improperly stored chow may substantially affect and alter the outcomes of known lung disease models. Changes in cell types, activatability of these cells, and increased numbers of cytokines with LPS stimulation showed a clear signal of an increased level of neutrophils in the airways and increased certain cytokine levels compared with control. This has also been shown in human studies of asthma associated with obesity, in which the predominant cells present in lavageable airspaces are neutrophils. Because the only difference between the chows used in our studies were the fat components and the time, they were allowed to be unrefrigerated, we conclude that lipid alteration, in particular oxidation, is a significant contributor. Further supporting this conclusion is the positive correlation between LPS‐induced airspace neutrophil counts with chow and plasma MDA levels. This has major implications on the way we interpret obese vs lean data in injured lung models. It also sets the stage for further understanding how diet affects out innate immunity, pulmonary microenvironment, surfactant changes, and endless other functions that alter the course of pulmonary disease.

## AUTHOR CONTRIBUTIONS

Marta Kokoszynska, Anne E. Dixon, Matthew E. Poynter, and Benjamin T. Suratt contributed to the concept and design of the study. Marta Kokoszynska, Niki D. Ubags, Joseph J. Bivona, Sebastian Ventrone, Leah F. Reed, Matthew J. Wargo, and Matthew E. Poynter were responsible for collection and analysis of experimental data. All authors were involved in the drafting and editing of the manuscript.
